# Deficiency of multiple RNA silencing-associated genes may contribute to the increased susceptibility of *Nicotiana benthamiana* to viruses

**DOI:** 10.1007/s00299-024-03262-3

**Published:** 2024-06-19

**Authors:** Márta Ludman, Schamberger Anita, Károly Fátyol

**Affiliations:** 1 Institute of Genetics and Biotechnology, Hungarian University of Agriculture and Life Sciences, Szent-Györgyi A0020U 4, Gödöllő, 2100 Hungary; 2 Institute of Enzymology, HUN-REN Research Centre for Natural Sciences, Magyar Tudósok Körútja 2, Budapest, 1117 Hungary

**Keywords:** Plant virus, RNA silencing, *Nicotiana benthamiana*

## Abstract

Recently published high-quality reference genome assemblies indicate that, in addition to *RDR1*-deficiency, the loss of several key RNA silencing-associated genes may contribute to the hypersusceptibility of *Nicotiana benthamiana* to viruses.

**Supplementary Information:**

The online version contains supplementary material available at 10.1007/s00299-024-03262-3.

Antiviral RNA silencing is the primary defense measure against viruses in plants (Lopez-Gomollon and Baulcombe 2022). Its main steps have been clarified in recent years and can be summarized as follows. The pathway is triggered by virus-derived double-stranded RNAs (dsRNAs) produced by virus-encoded RNA-dependent RNA polymerases (RDRs). These dsRNAs are processed by RNase III-like enzymes (DCLs), resulting in 21–24 nt long primary viral small interfering RNA duplexes (vsiRNAs). The process is assisted by double-stranded RNA-binding proteins such as DRB4 and possibly DRB3 and DRB2 (Qu et al. 2008; Raja et al. 2014; Barton et al. 2017; Fátyol et al. 2020; Incarbone et al. 2021). RNA silencing is typically accompanied by an amplification step catalyzed by host-encoded RDRs (mainly RDR1 and RDR6), which synthesize dsRNAs using aberrant single-stranded (ss) viral RNAs as templates. These dsRNAs are also processed by DCL-DRB complexes, yielding secondary vsiRNA duplexes. Eventually, one strand of the vsiRNA duplex is incorporated into an Argonaute (AGO) protein, resulting in an RNA-induced silencing complex (RISC). Antiviral RISCs are able to limit the replication of invading viruses in a sequence-specific manner by RNA cleavage or translational repression. As a highly effective countermeasure however, viruses produce proteins that, in addition to their canonical roles in the viral life cycle, are capable of suppressing antiviral RNA silencing in various steps (viral suppressors of RNA silencing, VSRs) (Ding 2023).

Studies based on the extensive genetic resources of the model plant *Arabidopsis thaliana* have played an instrumental role in clarifying the details of antiviral RNA silencing. Additionally, research using the LAB strain of *Nicotiana benthamiana* has contributed greatly to understanding the molecular arms race between plants and viruses (Bally et al. 2018). This strain has a 72 bp insertion in its *RDR1* gene, that prematurely terminates the gene’s open reading frame. It is generally assumed that this dysfunction affects the plant’s response to viral infection, and most importantly, makes the plant susceptible to more than 500 different viruses. Curiously, however, Ying et al. found that supplementing the LAB strain with *RDR1* from *Nicotiana tabacum* does not lead to increased resistance, but rather enhances virus infection (Ying et al. 2010). Thus, the authors suggested that *RDR1* could be a negative regulator of *RDR6* and that its inactivation in the LAB strain was likely due to high viral pressure to enhance *RDR6*'s antiviral role. In the latter scenario, *RDR1* deactivation is a consequence rather than a cause of the plant’s greater susceptibility to viruses. Either way, the findings of two recent studies reporting high-quality chromosome-level reference genome assemblies of *N. benthamiana* and *N. tabacum* are compatible with both hypotheses (Ranakawa et al. 2023; Wang et al. 2024). Comparative analysis of the two genomes revealed, that in addition to having a previously reported defect in *RDR1*, several additional key silencing genes were lost from one or both subgenomes of *N. benthamiana*, while both homeologs were retained in *N. tabacum*. The purpose of this Focus article is to link these findings to other recent studies on *N. benthamiana*’s RNA silencing-associated genes.

Secondary vsiRNAs are key to effective antiviral protection. Due to the aforementioned mutation of the *RDR1* gene, their production in the LAB strain of *N. benthamiana* is *RDR6*-dependent. The function of *RDR6* in *N. benthamiana* has been studied primarily in a plant line, in which the gene was silenced with a short-hairpin RNA (Schwach et al. 2005). Although the use of such a system provides invaluable information about gene function in many contexts, its application to study host-virus interactions can be problematic because viruses use various measures to compromise RNA silencing, which can also reduce the effectiveness of gene knock-down. To circumvent this potential problem, previously we created a *bona fide rdr6* mutant of *N. benthamiana*, using CRISPR/Cas9 genome editing (Ludman and Fátyol 2019). In the plant’s genome two potential *RDR6*-like ORFs could be identified. However, our results demonstrated that one of these alone can provide the RDR6 activity necessary for secondary vsiRNA production. These findings are consistent with the two reports mentioned above, which show that the genome of *N. benthamiana* contains a single functional *RDR6* gene and an *RDR6* pseudogene (Ranakawa et al. 2023; Wang et al. 2024).

In addition to regulating host gene expression *AGO1* has been consistently implicated in antiviral protection (Silva-Martins et al. 2020). In the new reports four *AGO1* homeologues—two *AGO1A* (*Nbe10g25940* and *Nbe02g25990*) and two *AGO1B* (*Nbe06g21200* and *Nbe05g23300*)—were annotated in the *N. benthamiana* genome, a number lower than that in *N. tabacum*, which has six of them (Ranakawa et al. 2023; Wang et al. 2024). The functions of the *N. benthamiana AGO1* homeologues were previously studied in plants, where they were selectively inactivated using CRISPR/Cas9 (Ludman and Fátyol 2021). Interestingly, inactivation of only one of the *AGO1A* or *AGO1B* homeologues was sufficient to produce distinct phenotypes in the plants. The *ago1a* mutants developed normally but were hypersusceptible to TCV infection. AGO1B deficiency, however, was incompatible with normal development as homozygous *ago1b* plants could not be obtained, indicating embryonic lethality. Even heterozygotes exhibited severe leaf malformations and were almost completely sterile. Nevertheless, these heterozygotes were hypersusceptible to TCV infection, indicating that *AGO1B* is also involved in antiviral protection. The two newly annotated *AGO1* homeologues bear high overall similarity to those previously inactivated by genome editing, but both carry deletions in their N-terminal domains (Fig. S1). Although less conserved than the rest of the protein, the N terminal region of AGOs is implicated in a number of important functions (Martín-Merchán et al. 2023). Whether the aforementioned deletions affect the activities of the two newly annotated *AGO1* homeologues remains to be determined.

*AGO2* is involved in various stress responses in plants, including antiviral protection. This was conclusively proven more than 10 years ago by demonstrating that the *ago2* mutant *A. thaliana* is hypersusceptible to TCV and CMV infections (Harvey et al. 2011). Later, the critical role of the *N. benthamiana AGO2* orthologue in limiting virus infections was also corroborated using genome-edited *ago2* mutant plants (Ludman et al. 2017). Inactivating two alleles of a single *AGO2* gene increased the susceptibility of the plant to a number of different viruses, providing evidence for the existence of one functional *AGO2* gene, despite the plant's allotetraploid genome. This finding has now been also confirmed by the papers of Wang et al. (2024) and Ranakawa et al. (2023).

The investigation of the antiviral role of *N. benthamiana AGO5* has been the subject of two recent reports, both of which employed CRISPR/Cas9 genome editing to inactivate the gene (Ludman et al. 2023; Tu et al. 2023). The *AGO5* gene (*Nbe10g12070* in Wang et al. 2024) encoding the 978 aa long AGO5 protein was inactivated in both studies, resulting in increased susceptibility to several viruses. Consistent with the allotetraploid nature of *N. benthamiana*, a second related *AGO5*-like ORF (*Nbe02g30110* in Wang et al. 2024) can also be retrieved from the plant’s genome. This ORF encodes a protein of 919 aa, which overall exhibits 86.6% identity and 88% similarity to the AGO5 protein encoded by *Nbe10g12070*. Importantly however, this AGO5 variant carries a deletion that removes the N-terminal 56 aa segment of its PIWI domain, including the first catalytically essential aspartic acid (Fig. S2). Consequently, this protein is most likely dysfunctional, which is consistent with the finding that a mutation in *Nbe10g12070* alone is sufficient to increase the plant’s susceptibility to viral infections.

In addition to the silencing-related genes discussed thus far, one copy of each of *DCL2 DCL3* and *DRB4* genes present in *N. tabacum* has been lost in *N. benthamiana*, and the number of *AGO4*-like genes is also lower in this species than in *N. tabacum* (Ranakawa et al. 2023; Wang et al. 2024). Because the hierarchical actions of multiple AGOs relying on both primary and secondary vsiRNAs, are needed to establish potent antiviral resistance (Ludman et al. 2023), all of the above deficiencies conceivably contribute to *N. benthamiana*’s hypersusceptibility to viruses (Fig. 1). In this context, an interesting question arises as to why during the evolution of *N. benthamiana*, genes associated with RNA silencing appeared to have been preferentially lost. Although there is no definitive answer yet, based on existing data, two non-exclusive hypotheses can be formulated as possible explanations. In the *Suaveolentes* section of the genus *Nicotiana*, to which *N. benthamiana* belongs, allopolyploidization was likely key to adapting to Australia's harshest climatic and ecological regions (Ranakawa et al. 2023). RNA silencing appears to be particularly important in relation to the genomic instability that often accompanies allopolyploid hybridization, both as cause and effect (Comai 2000). Alternatively, or perhaps complementarily to the above, it has also been suggested that impairing the plant’s viral defenses leads to metabolic changes that promotes early vigor and, consequently, more successful adaptation to adverse environmental conditions (Bally et al. 2015). Although the details are obviously sketchy, both of the above could have significant but difficult to predict consequences for the evolution of the plant’s RNA silencing apparatus.

**Fig. 1:**
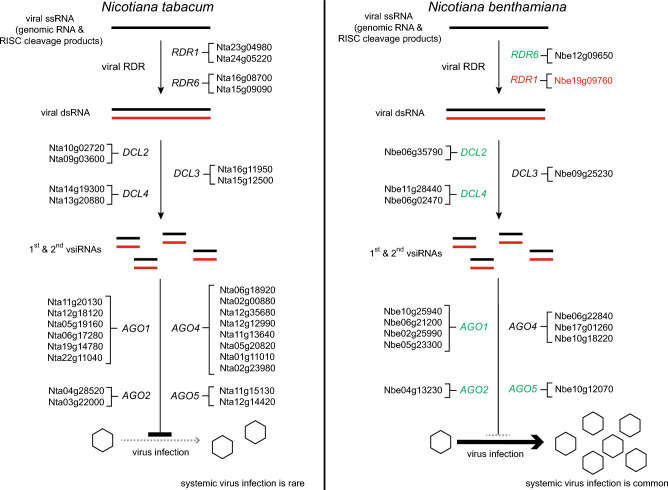
Annotated homeologues of key antiviral RNA silencing genes in the *N. tabacum* and *N. benthamiana* genomes. The genes were placed within the framework of the canonical antiviral RNA silencing pathway. Known pseudogenes were omitted. The gene identifiers used are from Wang et al. 2024. The *RDR1* gene of the widely used LAB strain of *N. benthamiana* carries an inactivating 72 bp insertion and is therefore considered pseudogene in this strain (highlighted in red). *N. benthamiana*’s antiviral silencing genes, for which mutants have already been generated through genome editing, are highlighted in green (color figure online)

In any case, the impaired silencing is at least one of the factors that underlies *N. benthamiana*’s unparalleled susceptibility to viruses, thereby making it an exceptional model system for studying their interactions with the host. Additionally, in recent years, *N. benthamiana* has increasingly become the manufacturing platform of choice for molecular pharming. Among other factors, weak RNA silencing, which allows for efficient protein production, also contributes to the plant’s popularity. Further improvement of this and other beneficial properties of *N. benthamiana* can now rely not only on rapidly evolving genome editing technologies but also on the genomic resources reported in the studies of Wang et al. (2024) and Ranakawa et al. (2023).

### Supplementary Information

Below is the link to the electronic supplementary material.

Supplementary file1 (PDF 2380 KB)

## Data Availability

All data supporting the findings of this study are available within the paper and within its supplementary materials published online.
