# Supplementary material for: Deficiency of multiple RNA silencing-associated genes may contribute to the increased susceptibility of Nicotiana benthamiana to viruses
Source: Plant Cell Rep. 2024 Jun 19;43(7):177. doi: 10.1007/s00299-024-03262-3 (PMC11186921; doi:10.1007/s00299-024-03262-3)

DEFICIENCY OF MULTIPLE RNA SILENCING-ASSOCIATED GENES MAY CONTRIBUTE TO THE INCREASED SUSCEPTIBILITY OF NICOTIANA BENTHAMIANA TO VIRUSES

Márta Ludman, Schamberger Anita, Károly Fátyol

SUPPLEMENTARY FIGURE LEGENDS

Figure S1.

A Multiple sequence alignment of *N. benthamiana* AGO1A proteins: (1) AGO1A from Ludman and Fátyol 2021; (2) *Nbe10g25940* from Wang et al. 2023; (3) *Nbe02g25990* from Wang et al. 2023.

B Multiple sequence alignment of *N. benthamiana* AGO1B proteins: (1) AGO1B from Ludman and Fátyol 2021; (2) *Nbe06g21200* from Wang et al. 2023; (3) *Nbe05g23300.1* from Wang et al. 2023; (4) *Nbe05g23300.2* from Wang et al. 2023. *Nbe05g23300.1* and *Nbe05g23300.2* are alternatively spliced forms of the *Nbe05g23300* gene.

Figure S2.

Multiple sequence alignment of *N. benthamiana* AGO5 proteins: (1) AGO5 from Ludman et al. 2023; (2) AGO5 from Tu et al. 2023; (3) *Nbe10g12070* from Wang et al. 2023; (4) *Nbe02g30110* from Wang et al. 2023. The major functional domains of AGO5 are indicated by colored boxes. The four acidic amino acids forming the catalytic tetrad of the PIWI domain are framed and labeled with red diamonds.

Figure S1.

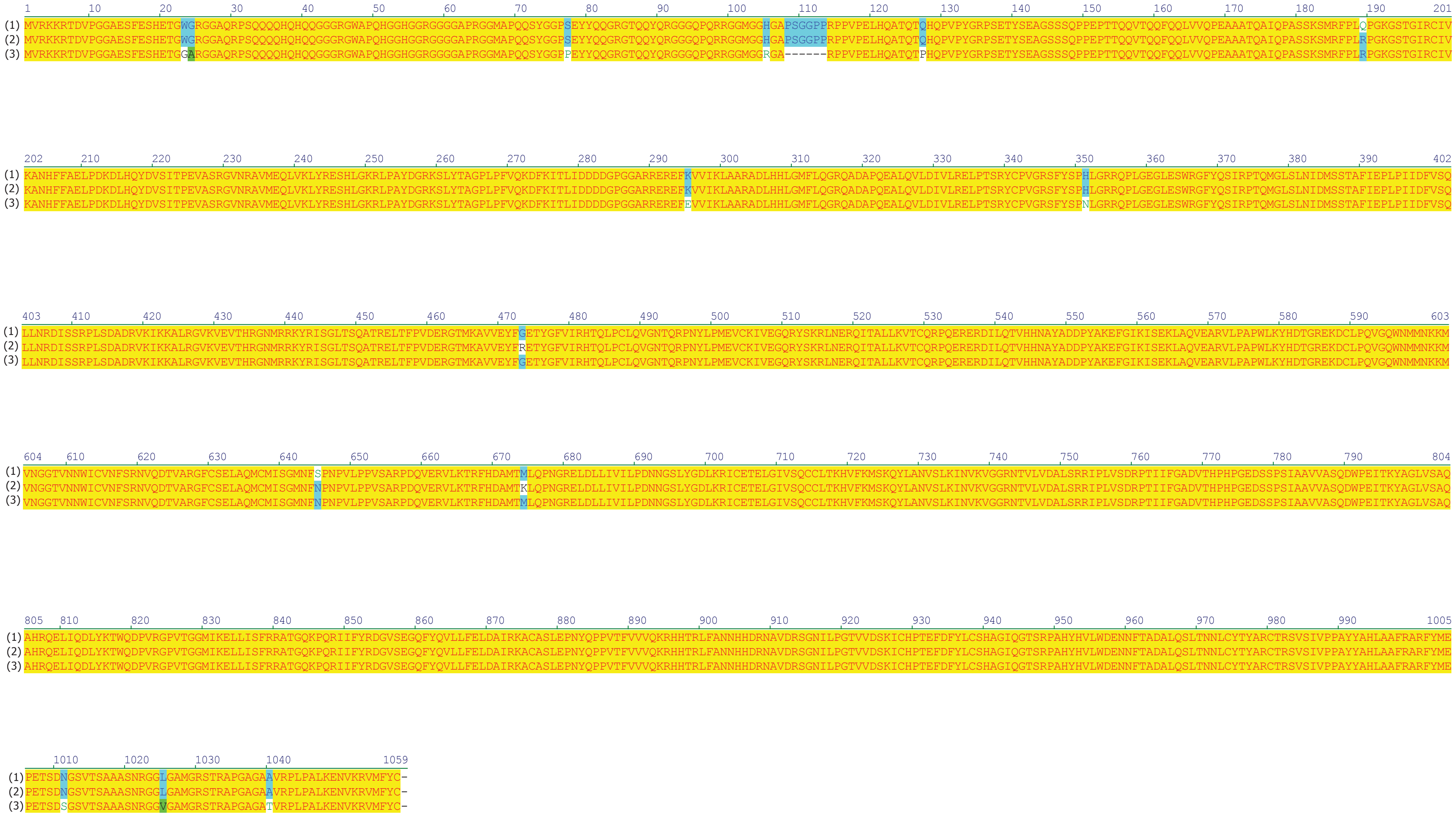

A

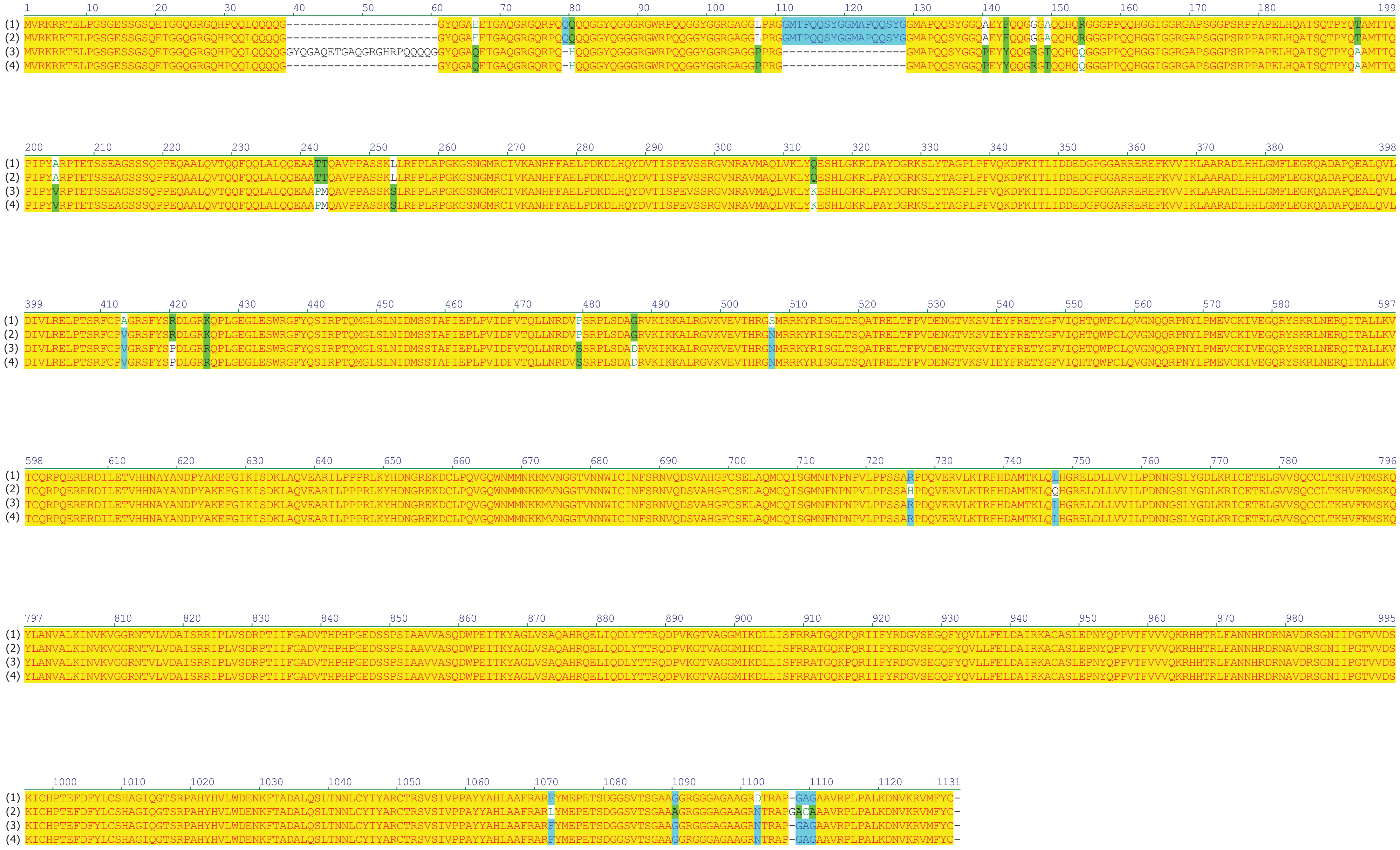

B

Figure S2.

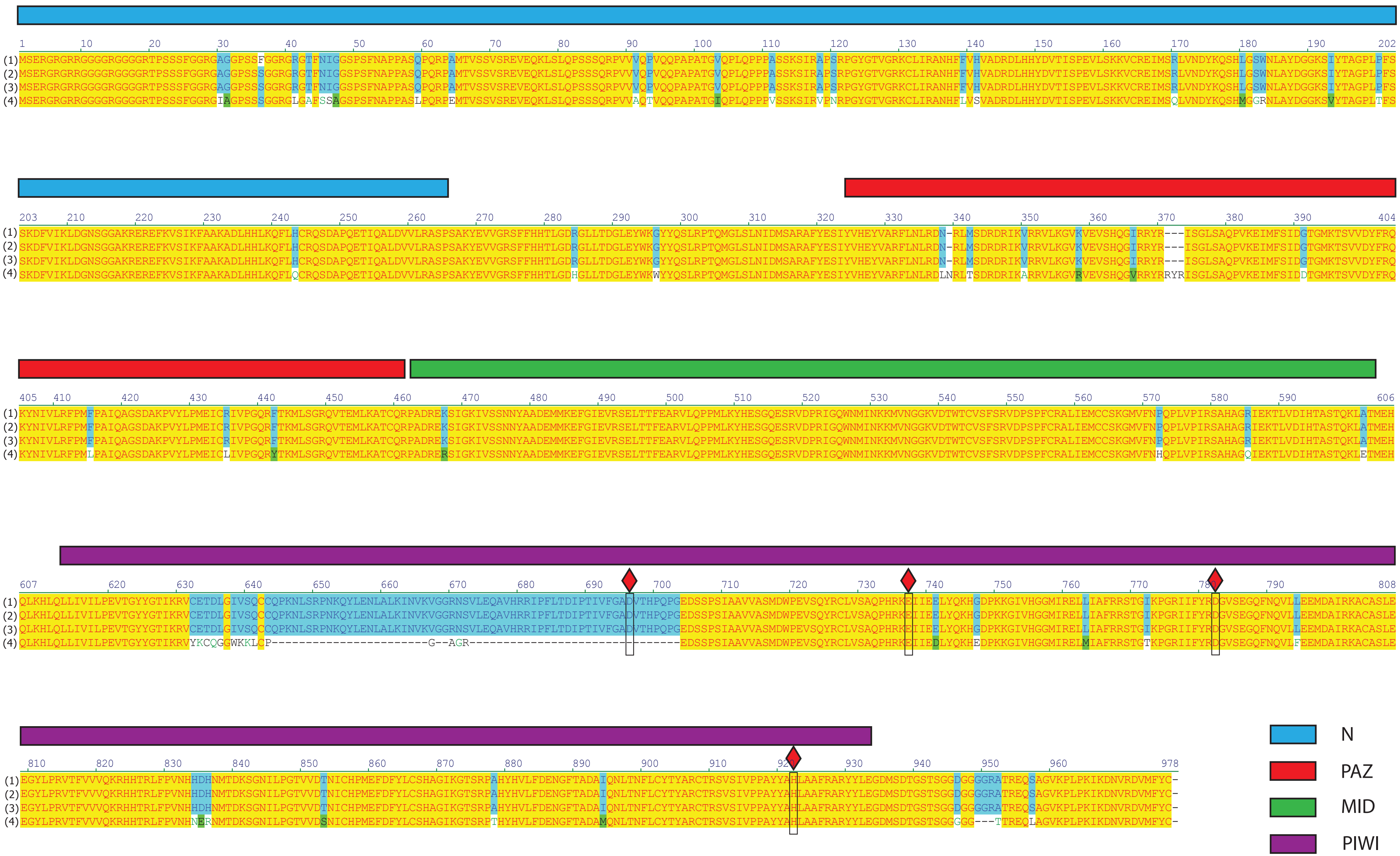

Supplement: Supplementary file 1 — Supplementary file1 (PDF 2380 KB) [file 299_2024_3262_MOESM1_ESM.pdf]
